# Solid pseudopapillary tumor of the uncinate process of the pancreas in a 9-year-old child

**DOI:** 10.1186/s43046-025-00307-w

**Published:** 2025-08-11

**Authors:** Dmitriy A. Pyhteev, Yuriy Yu. Sokolov, Leonid M. Elin, Yuriy N. Filyushkin, Marina O. Elina, Roman A. Akhmatov

**Affiliations:** 1 Moscow Regional Scientific Research Clinical Institute M.F. Vladimirsky, Moscow, Russian Federation; 2 Russian Medical Academy of Continuous Professional Education, Moscow, Russian Federation; 3 St. Vladimir Children’s Moscow Clinical Hospital, Moscow, Russian Federation; 4 Russian Children’s Clinical Hospital, Moscow, Russian Federation

**Keywords:** Pancreas, Pancreatic neoplasms, Child, Solid pseudopapillary tumor

## Abstract

**Background:**

Solid pseudopapillary tumor (SPT) is a rare tumor of the pancreas with a low degree of malignancy. This tumor is most common in the female field, and in the children’s population, this pathology is less common and occurs on average for the ages of 11–14 years old. The differential diagnosis of the tumor is difficult due to the lack of specific symptoms. The most informative methods of examination are computed tomography (CT) and magnetic resonance imaging (MRI). Surgical treatment is currently the most beneficial method of choice for the treatment of SPT.

Case presentation.

A 9-year-old girl for the first time experienced multiple episodes of vomiting and abdominal pain against the background of complete well-being. There were performed ultrasound examination, CT, and MRI, the results of which revealed a cystic neoplasm of the head of the pancreas. There was carried out a differential diagnosis with serous cystadenoma, mucosal cystadenoma, and SPT. The child underwent surgical intervention — upper-median laparotomic access and enucleation of the tumor of the hook-shaped process of the pancreas. The postoperative period proceeded smoothly. According to the histological examination, there was identified the solid-pseudopapillary tumor of the pancreas.

**Conclusion:**

The presented clinical case of tumor enucleation of a rare uncinate process tumor in a child is an alternative to radical surgical resections of the pancreas.

## Introduction

The solid pseudopapillary tumor (SPT), first described in 1959 by Frantz, is a rare tumor of the pancreas with a low degree of malignancy. The frequency of occurrence of SPT is from 2 to 3% of all cystic neoplasms of the pancreas and about 0.9% to 2.7% of all exocrine neoplasms of the pancreas [1, 2]. In 2000, on the basis of histological criteria by the World Health Organization, SPT was classified as a benign neoplasm with a low degree of malignancy. However, in 2010, it was reclassified as a malignant neoplasm with a low degree of malignancy [3]. SPT occurs mainly in females, and the average age is 21 years old [4]. In the pediatric population, SPT is rare and in most cases is detected at the age of 13–14 years old. The difficulty of detecting SPT is explained by the asymptomatic course and the nonspecific nature of the symptoms until the tumor increases in size or the inflammation occurs [5]. The method of choice for the treatment of this pathology is surgical resection with its various approaches, with a favorable outcome of healing > 95% [6]. The method of surgical aid depends on the localization of the tumor and the proximity to the Wirsung duct [7]. Despite the good results of the treatment, the cases of SPT relapse persist.

## Case report

A 9-year-old girl, against the background of complete well-being, for the first time experienced multiple episodes of vomiting and abdominal pain and therefore was hospitalized in a medical facility in the Moscow region There were performed such tests as ultrasound examination and computed tomography (CT), the results of which revealed a cystic neoplasm of the head of the pancreas.

For further examination and treatment, the child was transferred to the Moscow Regional Research and Clinical Institute n.a. M.F. Vladimirsky During the admission, her condition was of moderate severity. The appetite was reduced. There was noted some nausea, and the vomiting episodes were not repeated. The palpation of the abdomen in the epigastric region showed marked soreness without the symptoms of the peritoneum irritation.

**Fig. 1:**
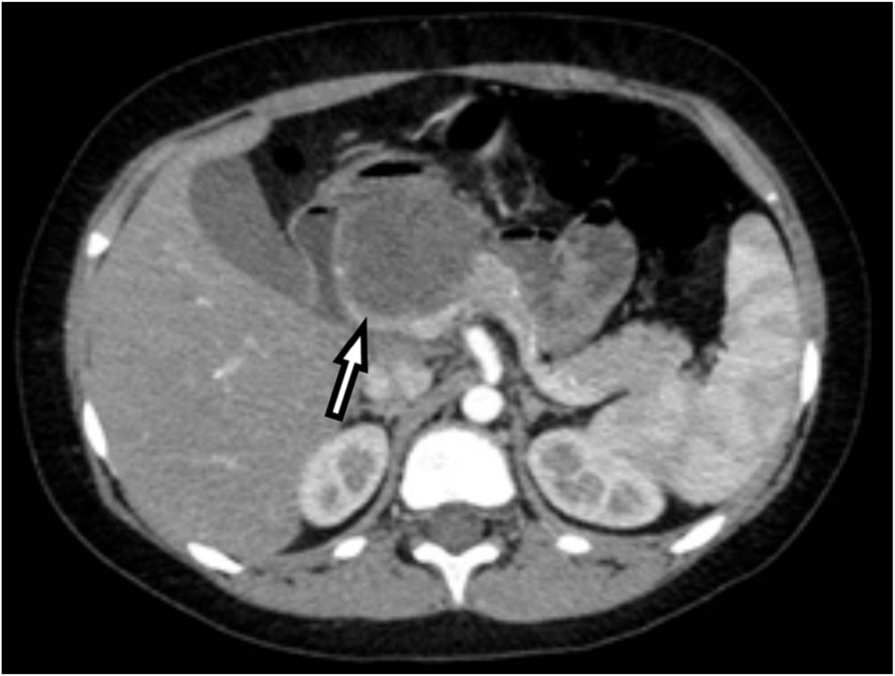
According to the CT results, an inhomogeneous hypodense formation was noted at the border of the head and the body of the pancreas, with uneven contours, 48 × 48 × 50 mm, and with contrast, there was no increase in blood flow inside the formation

**Fig. 2:**
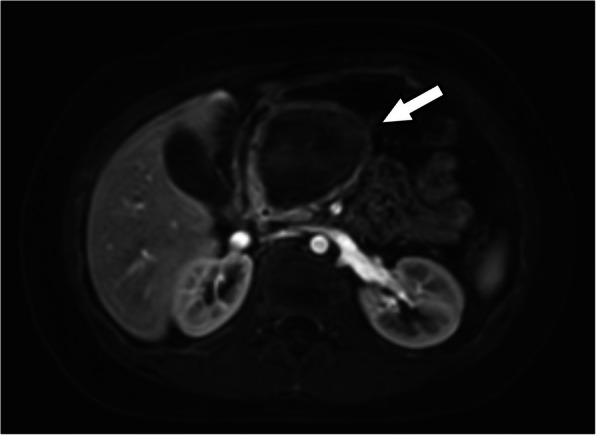
MRI with contrast. According to MRI data, there was visualized a heterogeneous cystic formation, 55 × 51 × 48 mm, at the level of the head and body of the pancreas

According to the laboratory tests, there was an increase in proinflammatory markers: leukocytosis 19.05^109, neutrophilic leukocytosis 74%, and an increase in C-reactive protein to 96.42 mg/l. Blood alpha amylase and urine diastasis were within the normal limits. In the study of cancer markers (CEA, CA 19–9, CA 15–3, CA-125, alpha-fetoprotein), there was no increase in indicators. According to the ultrasound data, the pancreas was not enlarged, with clear, even contours; a rounded formation with dense walls was visualized in the head area, measuring 48 × 47 × 48 mm; and with color duplex scanning, there was identified the increased blood flow into the walls and the absence of the tumor inside. According to the CT results, an inhomogeneous hypodense tumor was noted at the border of the head and body of the pancreas, with uneven contours, 48 × 48 × 50 mm in size, with the density of up to 35 units (Fig. 1). In CT, with contrast, there was no increase in blood flow inside the tumor. There was also an increase in the regional lymph nodes up to 7 mm. According to magnetic resonance imaging (MRI) data, there was visualized a heterogeneous cystic tumor, 55 × 51 × 48 mm, at the level of the head and the body of the pancreas (Fig. 2). The tumor anteriorly pushes back the pyloric part of the stomach, deforming the medial wall of the 12-duodenum; the lower contour merged with infiltrative changes around the truncus coeliacus; along the upper edge, it adhered to a. hepatica communis and a. lienalis, and along the medial edge, it adjoined v. mesenterica superior. There were also visualized multiple regional lymph nodes up to 7 mm in diameter. When contrasting, there was an uneven increase in the signal from the wall and no contrast inside the cyst lumen. There was also performed esophago-gastroduodenoscopy, according to the results of which there was revealed erythematous gastropathy. Taking into account the examination data, there was carried out the differential diagnosis with serous cystadenoma, mucosal cystadenoma, and SPT.

Due to the inflammatory changes, the child underwent antibacterial therapy (ampicillin/sulbactam) and antispasmodic therapy. Taking into account the relief of pain syndrome and dyspeptic disorders, the normalization of proinflammatory blood markers, the reduction of the size of the tumor, according to the ultrasound (45 × 45 × 44 mm), and the lack of data for the acute pancreatitis, the child was discharged under the supervision of a pediatric surgeon at the place of residence with subsequent hospitalization to decide on the further treatment tactics.

The child was rehospitalized in the Moscow Regional Research and Clinical Institute n.a. M.F. Vladimirsky pediatric surgical department after 26 days.

**Fig. 3:**
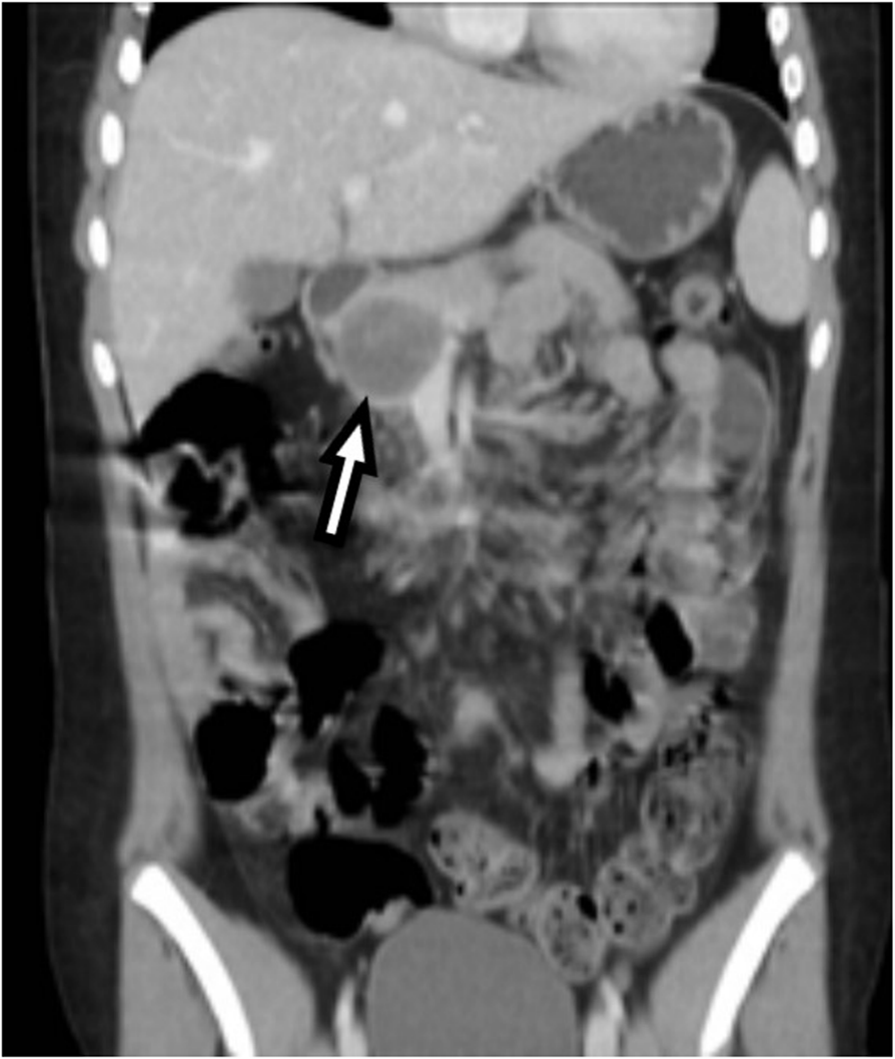
CT formation at the border of the head and body of the pancreas is a heterogeneous hypodense formation, with uneven internal contours, 37 × 38 × 35 mm in size, with a density of up to 40 units. HU, with contrast enhancement of blood flow, is not visualized

During the ultrasound, the pancreas is not enlarged, and a rounded tumor with dense walls, uneven density, and average echogenicity, size 48 × 36 × 38 mm, is visualized in the head area. According to CT data, the tumor at the border of the head and the body of the pancreas is an inhomogeneous hypodense tumor, with uneven internal contours, measuring 37 × 38 × 35 mm (Fig. 3). There was also an increase in regional lymph nodes up to 7 mm.

**Fig. 4:**
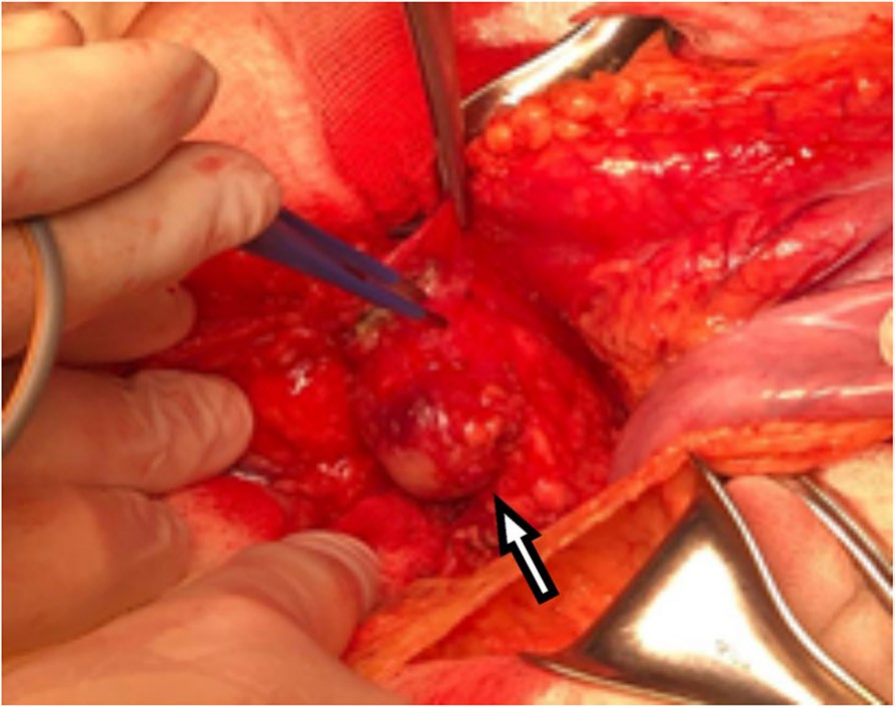
The formation of a dense consistency in the projection of the uncinate process of the pancreas

Taking into account the absence of an increase in proinflammatory markers, the normal indicators of cancer markers, and the results of radiation treatments, there was made a decision to perform surgery with upper-median laparotomy access. Intraoperatively, after opening the omentum bag and the posterior leaf of the peritoneum in the projection of the head in the area of the hook-shaped process of the pancreas on the anterior surface, there was revealed a dense consistency of the tumor with clear contours, size 37 × 38 mm (Fig. 4). As for the intraoperative period, the macroscopic picture was characterized by the absence of the enlarged lymph nodes.

There was performed the mobilization of the 12-duodenum by Kocher. There was no conflict of the tumor with the 12-duodenum and the vessels. The tumor was enucleated in a blunt and sharp way without any technical difficulties. There was no connection between the tumor with the main pancreatic duct (MPD) and the intake of the pancreatic juice. The operation was completed by draining the stuffing the box. There were no complications or blood loss.

The postoperative period proceeded smoothly. The drainage from the omentum bag was removed 4 days after the operation. There were no clinical and laboratory data on acute pancreatitis. The child was discharged from the hospital in a satisfactory condition on the 8 th day after the operation. According to the histological examination, there was diagnosed a solid pseudopapillary tumor of the pancreas.

## Discussion

Pancreatic SPT in children is a rare pathology with a low degree of malignancy compared to adults, accounting for 1%–2% of all pancreatic tumors [8]. In pediatric papulation, the most frequent age range of detection is 13–14 years [8, 9]. Due to the development of radiation research methods, the frequency of tumor detection increases. In the early stages of the disease, the clinical manifestations are practically absent [10]. However, as SPT grows, there could appear nonspecific symptoms, such as nausea, vomiting, bloating, palpable formation, fever, and pain caused by the compression of surrounding organs. Currently, there are no specific cancer markers and laboratory research methods for the verification of SPT [7, 10, 11]. Ultrasound is a subjective method of research, the diagnostic significance of which depends on the personal experience of the doctor; therefore, it is used as a screening method that has great accessibility in medical institutions and speed of execution. The main diagnostic methods at the preoperative stage are CT and MRI [6–8, 10]. The characteristic signs of SPT are encapsulated solid, cystic-solid, and cystic components, a lack of blood flow inside the tumor, and not infrequently with the signs of internal hemorrhages. CT scans determine a shadow of varying density, a “sign of a floating cloud.” It is also possible to assess the presence of invasion into the surrounding tissues, the enlargement of the lymph nodes, metastases, and vascular invasion with contrast enhancement, which is very important due to the tendency of the tumor to relapse [6–8, 10]. MRI, which has a higher resolution of tissues, in comparison with CT, has a higher informative value in assessing the relationship between the tumor with the bile ducts and the pancreatic ducts, which allows you to plan the course and the volume of the surgical aid more carefully [6, 10, 12]. Despite the wide awareness of SPT and the diagnostic significance of radiation research methods, in most cases, it is difficult to make the correct diagnosis at the preoperative stage. The differential diagnosis is complicated and is primarily stated with neuroendocrine tumors of the pancreas, serous cystadenoma, and mucinous cystadenoma. As for the fine needle biopsy with cytological examination, unfortunately, it is not always informative [1, 2, 10, 12]. The histological examination of the remote formation with immunohistochemistry is crucial for the diagnosis.

Considering that in the presented clinical example the child was 9 years old, and according to the results of the examination (ultrasound, CT, and MRI), we did not find the characteristic radiation signs for any pathology, and therefore, we could not establish an accurate diagnosis at the preoperative stage. Considering the fact that against the background of conservative therapy with repeated MRI after 1 month the tumor decreased by two times and the fact that there was also no solid component, the diagnosis of SPT was in doubt.

The frequency of occurrence of SPT is not high, so today there are no clear recommendations for the treatment of children with this pathology. Currently, the most preferred method of treatment is surgical removal of the tumor and metastatic lesion without performing routine lymphadenectomy [1, 2, 6, 9, 10, 12, 13]. Despite the fact that the SPT of the pancreas is a tumor with a low degree of malignancy, according to the literature 2.6%–3.5%, there is a local invasion or metastases [9, 12]. The complete resection of the tumor allows to achieve a high percentage of cure > 95%. When choosing a surgical treatment method, it is necessary to take into account the balance between the volume of resection and the postoperative complications. Depending on the location, in the connection with the ducts of the bile ducts, pancreatic ducts, invasion, and size, a wide range of surgical aids could be used: pancreato-duodenal resection, duodenum preserving resection of the pancreatic head, distal pancreatic resection, total pancreatectomy, resection of the middle segment of PD, and enucleation.

Taking into account the accumulated experience in the treatment of SPT, the frequency of parenchymal-preserving operations has increased in recent years, allowing to preserve the exocrine and endocrine function of the pancreas in the almost full volume [13]. Wang and co-authors, when comparing traditional pancreatectomy and enucleation, noted that when performing enucleation, the operation time and the blood loss are significantly reduced, as well as the fact that there could be noted a lower frequency of exo- and endocrine insufficiency and a comparable recurrence rate [13]. Gao Y. and coauthors, analyzing their experience in treating 194 adult patients with SPT, also noted the advantage of enucleation in comparison with traditional pancreatectomy [13]. It is worth noting that enucleation is not possible in every case, and when the tumor comes into contact with the MPD, there is a high risk of traumatization of the latter with the development of pancreatic fistula and erosive bleeding in the postoperative period. In the presented case, the cystic tumor was located in the projection of the head of the pancreas, and given the close location to the MPD according to the results of radiation methods of investigation at the preoperative stage, it was impossible to exclude the expansion of the volume of the operation to duodenum-preserving resection of the head of the pancreas. In this connection, it was decided to refrain from laparoscopic access. Intraoperatively, during the pancreas revision, the tumor was of a dense consistency (solid) and was located in the hook-shaped process. When performing enucleation, we did not identify the connection with the pancreatic ducts, and there was no pancreatic juice, which allowed us to avoid such a traumatic operation as pancreatectomy in its various modifications and preserve the exocrine and endocrine function of the pancreas in a 9-year-old child.

## Conclusion

This study suggests that SPT enucleation, on condition there is no tumor connection with the pancreatic ducts, is a feasible and effective procedure in children, offering a safe alternative to radical surgical resections such as pancreaticoduodenectomy.

## Data Availability

No datasets were generated or analysed during the current study.

## Abbreviations

SPT: Solid pseudopapillary tumor
CT: Computed tomography
MRI: Magnetic resonance imaging
MPD: Main pancreatic duct
